# Selective Direct Laser Writing of Pyrolytic Carbon Microelectrodes in Absorber-Modified SU-8

**DOI:** 10.3390/mi12050564

**Published:** 2021-05-17

**Authors:** Emil Ludvigsen, Nina Ritter Pedersen, Xiaolong Zhu, Rodolphe Marie, David M. A. Mackenzie, Jenny Emnéus, Dirch Hjorth Petersen, Anders Kristensen, Stephan Sylvest Keller

**Affiliations:** 1 National Centre for Nano Fabrication and Characterization, DTU Nanolab, Technical University of Denmark, Ørsteds Plads, Building 345B, 2800 Kgs. Lyngby, Denmark; emillu@dtu.dk (E.L.); ninafossdalritter@gmail.com (N.R.P.); 2 Department of Health Technology, DTU Health Tech, Technical University of Denmark, Ørsteds Plads, Building 345C, 2800 Kgs. Lyngby, Denmark; xizhu@dtu.dk (X.Z.); rcwm@dtu.dk (R.M.); akri@dtu.dk (A.K.); 3 Department of Physics, DTU Physics, Technical University of Denmark, Fysikvej, Building 311, 2800 Kgs. Lyngby, Denmark; david.ma.mackenzie@gmail.com; 4 Department of Biotechnology and Biomedicine, DTU Bioengineering, Technical University of Denmark, Produktionstorvet, Building 423, 2800 Kgs. Lyngby, Denmark; jemn@dtu.dk; 5 Department of Energy Conversion and Storage, DTU Energy, Technical University of Denmark, Fysikvej, Building 310, 2800 Kgs. Lyngby, Denmark; dhpe@dtu.dk

**Keywords:** SU-8, pyrolysis, laser pyrolysis, direct laser writing, carbon, microelectrodes

## Abstract

Pyrolytic carbon microelectrodes (PCMEs) are a promising alternative to their conventional metallic counterparts for various applications. Thus, methods for the simple and inexpensive patterning of PCMEs are highly sought after. Here, we demonstrate the fabrication of PCMEs through the selective pyrolysis of SU-8 photoresist by irradiation with a low-power, 806 nm, continuous wave, semiconductor-diode laser. The SU-8 was modified by adding Pro-Jet 800NP (FujiFilm) in order to ensure absorbance in the 800 nm range. The SU-8 precursor with absorber was successfully converted into pyrolytic carbon upon laser irradiation, which was not possible without an absorber. We demonstrated that the local laser pyrolysis (LLP) process in an inert nitrogen atmosphere with higher laser power and lower scan speed resulted in higher electrical conductance. The maximum conductivity achieved for a laser-pyrolyzed line was 14.2 ± 3.3 S/cm, with a line width and thickness of 28.3 ± 2.9 µm and 6.0 ± 1.0 µm, respectively, while the narrowest conductive line was just 13.5 ± 0.4 µm wide and 4.9 ± 0.5 µm thick. The LLP process seemed to be self-limiting, as multiple repetitive laser scans did not alter the properties of the carbonized lines. The direct laser writing of adjacent lines with an insulating gap down to ≤5 µm was achieved. Finally, multiple lines were seamlessly joined and intersected, enabling the writing of more complex designs with branching electrodes and the porosity of the carbon lines could be controlled by the scan speed.

## 1. Introduction

Carbon is cheap, abundant, and biocompatible, has a high chemical stability, and has a wide potential window, which makes it an excellent electrode material for electrochemistry [1,2,3]. Hence, extensive research has been conducted in the field of carbon electrodes for various applications such as strain gauges in micro-electro-mechanical systems (MEMS) [4,5,6], electrochemical sensors [1,7,8], and energy storage devices [9,10,11,12,13]. Screen printing of carbon electrodes is arguably the cheapest and most widely used technique for the fabrication of 2D carbon electrodes for electrochemistry [14,15]. However, for many applications, a reduction of the electrode dimensions to the micro- and nanoscale is advantageous [16], which is difficult to achieve with screen printing [15].

Several approaches for the microfabrication of biocompatible carbon microelectrodes have been investigated, such as inkjet printing [17], UV lithography [18,19], plasma etching [20,21], and UV embossing [22]. Most of these strategies are based on the so-called carbon MEMS (CMEMS) process [23]. For carbon electrodes made by CMEMS and UV lithography, the desired electrode structure is defined by UV patterning of a photoresist followed by thermal treatment in a pyrolysis furnace [3,24]. There, it is heated to temperatures usually above 900 °C in an inert atmosphere for several hours to transform the photoresist into carbon through pyrolysis [24,25]. This approach usually provides excellent dimensional control and the possibility to tailor the electrode properties by the optimization of the pyrolysis process parameters [24]. However, it is a lengthy fabrication process with a high energy budget and it requires that all materials are compatible with high temperatures, limiting the choice of carrier substrates to materials such as silicon or fused silica.

Laser pyrolysis has been proposed as a novel method for the patterning of carbon microelectrodes [26,27]. The underlying mechanism for laser pyrolysis is that when the photons are absorbed by the polymer, the photonic energy is converted into heat [28]. Due to the high intensity of the laser beam and the relatively poor thermal conductivity of the polymer, the result is extremely rapid and highly localized heating, which results in a very high temperature gradient and a small heat-affected zone (HAZ) [10,28,29]. Through the local, thermal evaporation of non-carbonic species from the polymer, a conductive carbon trace is left behind in the path of the laser beam. Compared to the typical CMEMS process, laser pyrolysis allows for the fast patterning and rapid prototyping of electrodes. As a main advantage, laser processing avoids the treatment in the high temperature furnace, eliminating the requirement for a substrate compatible with high temperatures and allowing for the selective pyrolysis of polymer precursors [30,31].

The type and degree of disorder of the resulting carbon bonds depend on the precursor and lasing conditions [5,6,27]. Most laser pyrolysis studies have been conducted on polyimide (PI) films, which are commercially sold as Kapton^®^ [10,27,29]. Tour et al. have conducted extensive research on the laser pyrolysis of other polymers and carbon precursors demonstrating laser-induced graphene (LIG) on substrates such as potatoes [30], wood [32], cloth [30], cellulose [30], polystyrene [30], and polysulfone [33]. Laser pyrolysis of photoresist has so far only been demonstrated by Kostecki et al. [3]. The local laser pyrolysis (LLP) process yielded results similar to those achieved by furnace pyrolysis at 1000 °C [34]. The typical lasers used in LLP are pulsed gas- or solid-state lasers.

In this work, we add an absorber to the polymer precursor to ensure absorption at a specific wavelength. This allows the use of a commercially available, low-power, continuous wave (CW), semiconductor diode laser. This laser has the advantage of being compact and highly power efficient, with a very well-defined wavelength and a relatively small spot size, which should aid in achieving a smaller HAZ and a higher resolution. Furthermore, the addition of the absorber should allow for the selective pyrolysis of the absorber-modified precursor whilst an unmodified precursor and the underlying carrier substrate will remain unaffected by the laser. SU-8, an optically transparent, epoxy-based resin extensively used for micro- and nanofabrication [3,35], was chosen as the polymer precursor. When SU-8 is pyrolyzed at temperatures above 900 °C in an inert atmosphere, it is converted into pyrolytic carbon with excellent structural, electrical, and electrochemical properties [1,2,3,23]. However, SU-8 has not been previously used for LLP despite being an excellent precursor for pyrolysis. By combining the absorber with SU-8, we should be able to selectively and locally recreate the pyrolysis process using a low-power, near-infrared (NIR) laser. Thamdrup et al. demonstrated laser-induced local heating (LILH) of absorber-modified SU-8 for the thermophoretic manipulation of DNA [36]. Here, we explore and adapt this approach to directly write pyrolytic carbon electrodes in the SU-8, with excellent pattern control, high resolution, and a much lower overall thermal budget than furnace pyrolysis.

First, the effect of the absorber inclusion on the optical properties of the SU-8 was investigated. Then, the laser pyrolysis of the absorber-modified SU-8 was evaluated and optimized to achieve high conductivity and minimal line width of the resulting pyrolytic carbon. The chemical composition of the laser-written structures was characterized using Raman spectroscopy and XPS. Finally, the versatility and limitations of the process were evaluated.

## 2. Materials and Methods

### 2.1. Materials

Pro-Jet 800NP (a mixture of compounds from (dodecakis(ptolylthio)phthalocyaninato)copper(II) to (hexadecakis(ptolylthio)hthalocyaninato)copper(II) [37]) was purchased from FujiFilm Imaging Colorants Ltd. (Grangemouth, UK). Cyclopentanone was purchased from Sigma Aldrich (Darmstadt, Germany). SU-8 2035 was purchased from Kayaku Advanced Materials (Westborough, MA, USA). Carbon paste (Electric Paint) was purchased from Bare Conductive (London, UK).

### 2.2. Preparation of SU-8/Pro-Jet Films

**Figure 1:**
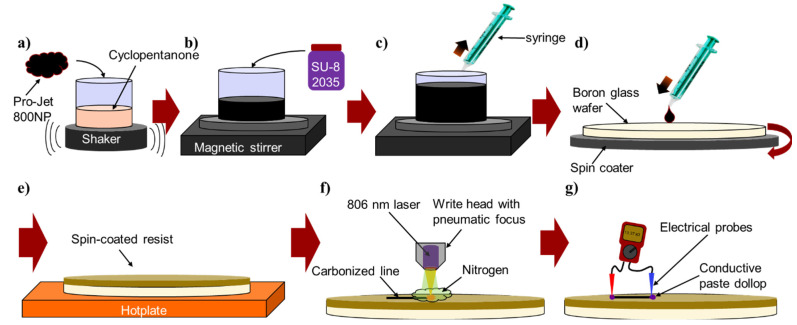
Overview of the preparation, exposure and characterization process: (**a**) absorber addition to solvent; (**b**) SU-8 addition; (**c**) stirring and sample collection; (**d**) sample deposition and spin coating; (**e**) solvent removal by baking on hotplate; (**f**) laser exposure in nitrogen atmosphere; (**g**) electrical characterization.

First, the absorber was added to the SU-8 photoresist as illustrated in Figure 1a–c. For this purpose, 0.12–4.04 g of Pro-Jet 800NP, which is highly absorbing in the red to NIR range, were dissolved in 10 mL cyclopentanone and agitated for 10 min. Then, 20 mL SU-8 2035 were added slowly using a polymer syringe while the solution was under constant stirring on a magnetic stirrer and the solution was left stirring until homogenized. Then, 4-inch boron glass wafers were dehydrated in an oven at 250 °C overnight. Approximately 4 mL of the homogenized SU-8/Pro-Jet solution were dispensed in the center of a wafer and spin-coated at 1000 rpm for 60 s with a ramping rate of 200 rpm/s on a programmable spin coater (Süss MicroTec RCD8 T spin coater, Süss MicroTec, Garching, Germany) (Figure 1d). Afterwards, the wafers were soft baked on a programmable hotplate (Harry Gestigkeit GmbH, Düsseldorf, Germany) at 75 °C for 30 min with a ramping rate of 2 °C/min (Figure 1e) and further dried in an oven at 90 °C for 24 h. The final absorber concentrations in the spin-coated SU-8 films varied from 0.0 to 11.09 wt % and the resist thickness was 13.9 ± 0.7 µm independently of the Pro-Jet concentration. The preparation of the SU-8/Pro-Jet films was performed in conditions with UV-filtered light, and no step for cross-linking of the SU-8 was included.

### 2.3. Optical Characterization of SU-8/Pro-Jet Films

Absorbance spectra of the boron glass wafers coated with SU-8 containing various concentrations of Pro-Jet were measured at normal incidence against air using a UV-VIS spectrophotometer (Shimadzu UV-2600, Shimadzu Corp., Kyoto, Japan). Absorbance spectra were recorded in the range from 200 to 1100 nm with 1.0 nm increments, and three separate measurements were conducted for each absorber concentration.

### 2.4. Direct Laser Writing

A modified mask-less aligner system (Heidelberg µPG 101IR, Heidelberg Instruments GmbH, Heidelberg, Germany) equipped with a continuous wave (CW) semiconductor-diode laser (Omicron BrixX 808–800HP, Omicron-Laserage Laserprodukte GmbH, Rodgau, Germany) was used for the direct laser writing of pyrolytic carbon electrodes (Figure 1f). The laser had a very narrow peak intensity at 806 nm wavelength and the system enabled scan speeds from 0.1 to 4.0 mm/s with powers varying from 1.0 to 800 mW. The laser had a Gaussian intensity profile, and the laser spot in the XY-plane was slightly elliptical with a horizontal focal diameter of 32.30 µm and a vertical focal diameter of 33.70 µm, giving a focal ellipticity of 1.04 at optimal focus. The focal diameters are defined by the radii from the center at which the intensity has dropped to 1/e^2^ = 13.53% of the peak intensity. Naturally, this will lead to higher intensity in the center compared to at the edge of the laser spot. Continuous control of the focus was achieved by the built-in pneumatic auto-focusing system. At the same time, the pneumatic focus system was used to control the atmosphere at the write-spot during direct laser writing. The laser exposures were performed in either ambient air or an inert N_2_ atmosphere. This was achieved by purging the laser spot with either compressed dry air (CDA) or N_2_ through the pneumatic focus nozzle in the write head. The gas flow rate through the write head was 3.5 L/min. Additionally, a second gas inlet and a ventilation outlet were installed in the chamber of the laser writing equipment, allowing for lateral purging and the removal of gaseous products. The desired exposure pattern was drawn in LayoutEditor (juspertor GmbH, Unterhaching, Germany) and read by the software controlling the laser before being written with the selected laser writing parameters. For comparison, a commercially available pulsed CO_2_-laser (Epilog Helix Mini 18, EpilogLaser, Golden, CO, USA) was also tested. The CO_2_-laser operated at 10.6 µm wavelength with a spot size of 100 µm in diameter and enabled powers from 0.3 to 30 W at scan speeds of 1–100 mm/s.

### 2.5. Chemical Analysis

The chemical composition of the laser written lines was studied using an X-ray photoelectron spectroscopy (XPS) system (K-Alpha, Thermo Fisher Scientific, Waltham, MA, USA) with the possibility of depth profiling by Argon (Ar) ion sputtering. Nine ion-milling cycles of 5 s each (3 keV ion energy, mid current, 2.0 mm gun raster width) were used for the in-depth analysis of the lines with an XPS measurement conducted after each cycle. A Raman spectrometer (Raman DXRxi, Thermo Fisher Scientific, Waltham, MA, USA) equipped with a laser operating at 532 nm wavelength was used to evaluate the presence and composition of carbon after the laser pyrolysis. Then, 500 µm × 500 µm maps of Raman spectra were obtained in the middle of a large, continuous, laser-written patch, using a sampling rate of 0.2 Hz and a laser power of 1 mW.

### 2.6. Structural Characterization

Optical microscopy (OM) imaging (Leica INM100 or Leica INM20, Leica Microsystems, Wetzlar, Germany) was employed to estimate the width *w* of the lines based on OM images of the blackened parts. The microscopes were connected to a computer running the NIS-Elements (Nikon, Tokyo, Japan) imaging software for live imaging.

SEM imaging (Zeiss Supra VP 40 or Hitachi TM3030 connected to a computer running the SmartSEM software, Carl Zeiss AG, Jena, Germany, or the TM3030Plus software, Hitachi High-Tech Global, Tokyo, Japan) of cleaved lines was used to determine the actual width *w* and thickness *h* of the pyrolyzed part for some selected lines.

Profilometry was employed to measure the depth of the grooves ablated by the laser during the direct laser writing. Both an optical profilometer (Olympus LEXT OLS4100, Olympus, Tokyo, Japan) and a stylus profilometer (Tencor Alpha Step IQ, KLA-Tencor, Milpitas, CA, USA) were used for verification. Four separate lines written with the same parameters were used for the groove depth measurements. The stylus scan parameters were 5 mm scan length, 1000 Hz sampling rate, and 100 µm/s scan speed, with a depth resolution of 0.1 µm. The stylus measurements were processed using the Alpha-step IQ software (KLA-Tencor, Milpitas, CA, USA) while data from the optical profilometer were processed with the SPIP ver. 6.7.8 software (Image Metrology, Hørsholm, Denmark). The groove depths were determined by measuring the average distance from the flat part on the top (outside the HAZ) to the lowest point of the trench. Another stylus profilometer (Dektak 150 Surface Profiler, Veeco Instruments Inc., Tucson, AZ, USA) was used to determine the final resist thickness after spin coating. The stylus scan parameters were as follows: 4 mm scan length, 130 µm/s scan speed, and 300 Hz sampling rate, giving a resolution of 0.4 µm. Three thickness measurements were conducted on each wafer on a small scratch exposing the glass substrate beneath.

### 2.7. Electrical Characterization of Carbon Lines

Electrical characterization of the laser written lines was done by placing a dollop of conductive carbon paste at either end of the line before measuring the resistance *R* through the line using a multimeter (Fluke 175 True RMS Digital Multimeter, Fluke, Everett, WA, USA) with two probes (Figure 1g). A probe station (EPS150 COAX, FormFactor GmbH, Thiendorf, Germany) fitted with wolfram needles and connected to a multimeter (Keithley 2000 Multimeter, Keithley Instruments, Cleveland, OH, USA) was used to confirm the resistance measurements obtained with the multimeter and the probes through the carbon paste dollops. The added contact resistance from the carbon paste was found to be completely negligible.

### 2.8. Estimation of Electrical Conductivity and Errors

The main parameter of interest for laser written electrodes is the conductivity σ given as

(1) $$\sigma =\frac{L}{RA}=\frac{4L}{R\pi wh}$$

where R is the measured resistance, L is the length, w is the width, and h is the thickness of a conducting line fabricated by laser writing. $A=\frac{1}{4}\pi wh$ is the cross-sectional area of the conducting line, assuming a semi-elliptical carbon cross-section. The actual length *L* of the lines between the dollops of conductive carbon paste was measured using OM. A constant value for *h* was assumed, cf. Section 2.6., in order to calculate an estimated conductance in cases where an actual thickness was not measured in cross-sectional SEM images.

The error bars on all graphs are computed either from the direct sample standard deviation or based on the law of error propagation,

(2) $$\delta_{q}=\sqrt{\overset{y}{\underset{i=1}{\sum }}{\left(\frac{\partial q}{\partial x_{i}}\delta x_{i}\right)}^{2}}\;$$

where $\delta_{q}$ is the combined error estimate used for the error bar and $\delta x_{i}=\sqrt{\frac{\sum^{}{\left(x_{i}-\bar{x_{i}}\right)}^{2}}{n}}$ is the sample standard deviation with $\bar{x_{i}}$ being the average of the $x_{i}$’th factor with *n* replicates.

### 2.9. Experimental Optimization of Local Laser Pyrolysis

We systematically evaluated process parameters influencing the direct laser writing of carbon electrodes: Pro-Jet content, atmosphere, laser power *P*, scan speed *v*, laser spot size *w_b_*, and number of scans *N* of the same line. The laser spot size for the laser system was constant at *w_b_* = 32.3 µm, while the other parameters were varied. At least four replicates of straight lines with a length of 5 mm and an inter-line spacing of 3 mm were written for a given set of parameters to be able to perform a statistical analysis. Afterwards, the written lines were evaluated structurally, chemically, and electrically as described above.

## 3. Results and Discussion

### 3.1. Optical Characterization of SU-8/Pro Jet Films

**Figure 2:**
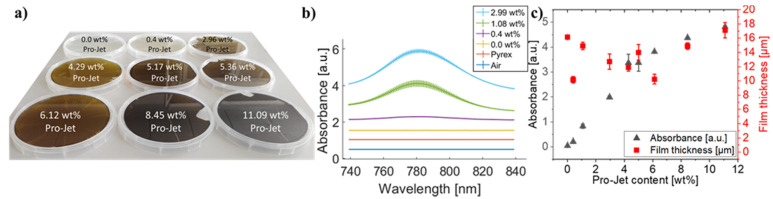
(**a**) Images of boron glass wafers coated with SU-8 containing different concentrations of Pro-Jet 800NP. (**b**) Absorption spectra recorded vs. air for the wafers coated with SU-8 containing the lowest investigated concentrations of Pro-Jet. The baselines have been artificially y-shifted to allow for better separation of the lines. (**c**) Extracted, average absorbances at 806 nm, which is the wavelength of the laser used for writing, and film thicknesses measured for each spin-coated Pro-Jet concentration. The error bars on (**b**,**c**) correspond to the standard deviation (*n* = 3).

First, the optical properties of SU-8 films modified with different absorber concentrations were determined. Figure 2 shows the influence of the addition of Pro-Jet 800NP on the absorption spectrum of SU-8 coated boron glass wafers. Clear opacity differences can be seen on the images of the wafers in Figure 2a, where the SU-8 films gradually become darker with increasing absorber concentrations. In Figure 2b, the recorded absorption spectra for the lowest Pro-Jet concentrations are shown. The measurements demonstrate that even Pro-Jet concentrations as low as 1% result in a distinct absorption peak at around 780 nm. The full absorbance spectrum for all concentrations is available in Figure S1 in the Supporting Information. As seen in Figure 2c, the absorption at 806 nm wavelength, corresponding to the wavelength of the laser used for direct writing, increased in an approximately logarithmic manner with increasing Pro-Jet concentration in the investigated range. For the direct laser writing in this study, the Pro-Jet content was fixed at 5.1–5.4 wt %, which was sufficient to ensure significant absorption of the laser beam while minimizing the influence of eventual local variations of absorber concentration in the SU-8 films.

### 3.2. Direct Laser Writing

**Figure 3:**
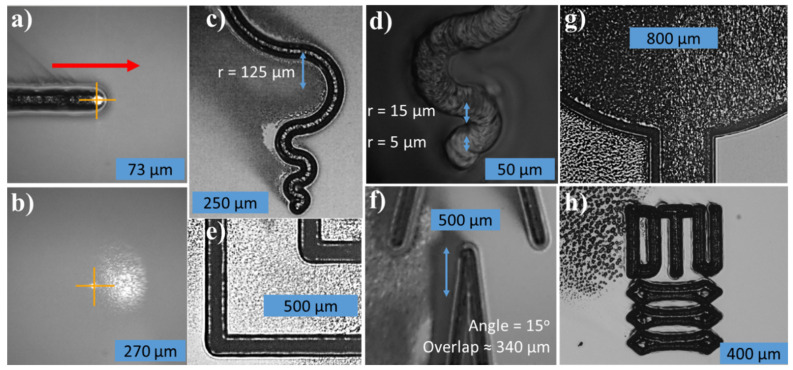
Direct laser writing in SU-8 with (**a**) and without (**b**) absorber. Both are written with 30 mW power and 0.5 mm/s scan speed. The red arrow on (**a**) indicates the writing direction. (**c**,**d**) Continuous serpentine lines written with decreasing radii of curvature (r). Lines written at 0.1 mm/s scan speed at (**c**) 30 mW and (**d**) 80 mW power. (**e**,**f**) Continuous lines written with a (**e**) 90° and (**f**) 15° angle. The lines were written with 80 mW power at (**e**) 2.0 mm/s and (**f**) 0.1 mm/s scan speed. (**g**) Filled circular patch, written by spacing lines closely together (≤10 µm spacing) using 80 mW laser power and 1.0 mm/s scan speed. (**h**) The logo of the Technical University of Denmark (DTU) written using 20 mW power and 0.3 mm/s scan speed.

The concept of direct laser writing with a modified mask-less aligner equipped with a CW laser operating at a wavelength of 806 nm was evaluated. Figure 3a,b presents images of laser writing in SU-8 with and without an absorber. The laser did not interact with SU-8 resin without an absorber. In comparison, a clearly visible line pattern is transferred into the resin modified with absorber. This demonstrates that pattern transfer is only possible due to the energy absorbed in the SU-8 film.

Figure 3c–h showcases the versatility and freedom of design of the direct laser writing by drawing intricate figures at various scan speeds and laser powers. Figure 3d,f show that the smallest resolvable radius of curvature was 15 µm and the smallest resolvable angle was about 15°, respectively. The “shadow” to the left of the line in e.g., Figure 3c is redeposited debris that was purged away from the writing spot by the pneumatic focus nozzle.

### 3.3. Chemical Analysis

**Figure 4:**
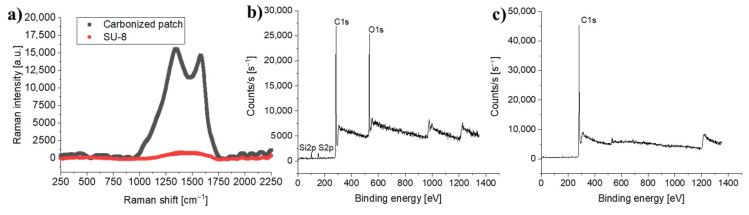
(**a**) Raman spectra of SU-8 (**red**) and laser-pyrolyzed carbon (**black**). The thickness of the lines corresponds to the standard deviation (*n* = 3). (**b**) XPS analysis of the surface of a carbonized line. (**c**) XPS analysis of a carbonized line after 45 s of ion milling.

XPS and Raman spectroscopy were used to analyze the composition of the blackened, laser-written lines, and to determine if the polymer had indeed been converted into pyrolytic carbon. Figure 4a shows a Raman spectrum with the disordered carbon band (D-band) located at 1346 cm^−1^ and the graphitic band (G-band) at 1567 cm^−1^. Similar spectra are typically observed for pyrolytic carbon obtained by pyrolysis of SU-8 resin in a furnace [24] as well as for carbon obtained by laser pyrolysis of PI [5,27,38]. The average intensity ratio of the D-band to the G-band (I_D_/I_G_-ratio) is 1.07 ± 0.01, which is similar to what has been reported for furnace pyrolyzed SU-8 at 1100 °C [24]. This demonstrates the presence of both graphitic and amorphous carbon regions [38,39]. Thus, Figure 4a confirms that direct laser writing in absorber-modified SU-8 was able to induce local pyrolysis and conversion of the photoresist precursor into pyrolytic carbon. The XPS spectra in Figure 4b,c further support the hypothesis that LLP converted the SU-8 into carbon. However, the surface layer of the carbon was oxidized. After a few cycles of Ar-ion sputtering, removing just a few nm of the top layer, the O_2_ content decreased dramatically (see Figure S2 in the Supporting Information).

### 3.4. Structural Characterization

**Figure 5:**
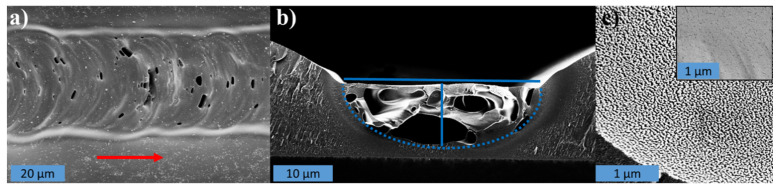
(**a**) Top view SEM image of a laser-written line. The red arrow indicates the writing direction. (**b**) Cross-sectional SEM image of the same line. The dotted blue line outlines the semi-elliptical cross-section of the pyrolyzed (carbonized) part and the solid blue lines indicate the thickness *h* and width *w*. (**c**) High-magnification micrograph of the top surface of the line revealing nanostructuring, the inset on (**c**) shows the surface of the non-carbonized SU-8 where no such nanostructuring is seen.

Figure 5a–c show SEM images of the top surface and cross-section of a laser-written, carbonized line. Figure 5a illustrates that semicircular scallops formed along the scan direction, following the melt front of the laser beam. Figure 5b shows that the cross-section of the carbonized, laser-written line is semi-elliptic and lies at the bottom of the groove. The actual width and thickness of the carbonized line was measured as indicated by the blue lines on Figure 5b. We hypothesize that the groove is caused by a combination of ablation and shrinkage of the photoresist precursor material. The latter is also observed for regular furnace pyrolysis of SU-8 due to the decomposition and evaporation of the polymer precursor [1,40]. It was found that the thickness *h* was more or less constant independently of the lasing parameters with *h* = 8.2 ± 2.1 µm. Data supporting this assumption of constant thickness can be found in Figure S3a,d in the Supporting Information. It was found that the line widths determined using the OM images consistently overestimated the actual width of the carbonized lines by a factor *F* = 4.17 ± 0.94 compared to the actual line widths measured by cross-sectional SEM imaging (see Figure S3b,c,e,f in the Supporting Information). However, width measurements of many hundreds of lines by SEM imaging were not viable. Therefore, the widths determined from the OM images were corrected accordingly using the correction factor *F*. The correction procedure is explained in detail in Section S3 in the supporting information. The depth of the ablated groove was also measured on these cross-sectional SEM images by measuring the vertical distance from the flat part of the SU-8 film outside the HAZ to the top of the carbonized part of the cleaved line in the groove. The close-up view in Figure 5c reveals that the carbon has a rough, nanostructured surface.

Three-dimensional (3D) profilometer scans (see Figure S4 in the Supporting Information) show that the groove is significantly deeper and the surface is rougher for pyrolyzed lines compared to non-pyrolyzed lines. The porous internal structure of the carbon visible in Figure 5b suggests that the elevated local temperature resulted in a substantial release of gasses and polymeric decomposition products. During laser writing, the material degasses, and the non-carbonaceous species are ablated. Finally, only carbon is left behind, similar to a furnace pyrolysis process [1]. This hypothesis is further substantiated by the Raman and XPS spectra in Figure 4.

### 3.5. Influence of Repetitive Laser Scans

**Figure 6:**
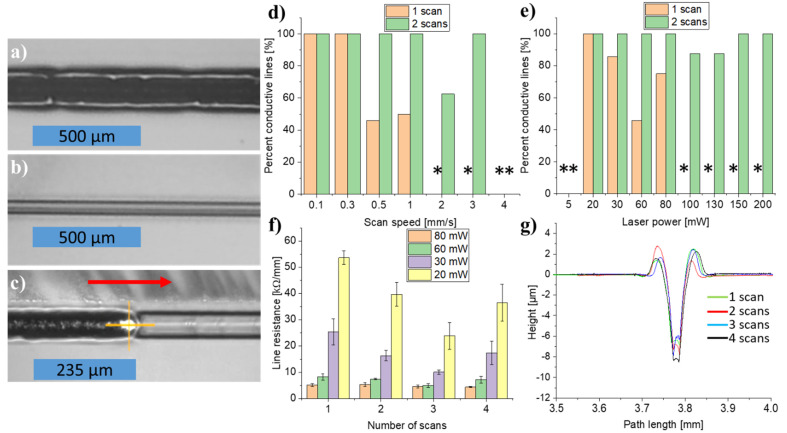
(**a**) Black, conductive line. (**b**) Grayish, non-conductive line. The lines in (**a**,**b**) have been written using 150 mW power, 0.5 mm/s scan speed, and one scan. (**c**) Second scan of a grayish line, converting it into black and conductive. The line was scanned at 30 mW power and 1.0 mm/s scan speed in both scans. The red arrow in (**c**) indicates the writing direction. (**d**,**e**) show the percentage of conductive lines (*n* ≥ 4) for 1 vs. 2 scans at different scan speeds (at 60 mW laser power) and laser powers (at 0.5 mm/scan speed), respectively. An asterisk (*) in place of a bar indicate a percentage of conductive lines equal to 0% for the given set of parameters. (**f**) Line resistance vs. number of scans at 0.5 mm/s scan speed for various laser powers. (**g**) Overlaid stylus profiles of lines scanned 1–4 times at 80 mW power and 0.5 mm/s scan speed. All lines were written under an N_2_ atmosphere.

It was observed that some of the written lines were black (Figure 6a), while other lines appeared grayish (Figure 6b). Some lines displayed both grayish and black regions with no systematic pattern. This effect was especially prominent for higher scan speeds and laser powers. Preliminary electrical measurements revealed that only the black lines were conductive. However, scanning the grayish line a second time with the laser as shown in Figure 6c converted the grayish regions into black and conductive ones. The hypothesis is that the black lines are pyrolytic carbon and that the grayish lines are merely the result of polymer ablation, where the heat induced by the absorbed photonic energy was insufficient to achieve full pyrolysis during the first scan. During the second scan of the line, the amount of absorbed energy is sufficient to complete the pyrolysis. As seen on Figure 6d,e, performing two scans instead of one considerably improved the total percentage of conductive lines and allowed for writing at faster scan speeds. An even higher number of scans did not further increase the percentage of conductive lines nor allow for writing at even higher scan speeds. In this study, a low-power, NIR CW laser is used. Compared to the use of pulsed lasers, CW lasers may experience an increased impact of the plume effect [28]. In particular at highser power, the plume formed during lasing due to material ablation and decomposition might effectively shield the writing spot from further interaction with the laser beam. When the scan speed and/or laser power is high and only one scan is used, it is likely that the plume has no time to disappear before the laser moves on, which may cause the observed interruptions and un-sustained pyrolysis of the lines.

Furthermore, it was investigated if repetitive laser scans were able to enhance the conductivity of already conductive lines. Figure 6f presents a slight decrease in line resistance for repetitive scans at low laser powers.

Figure 6g shows overlaid stylus profiles of lines scanned 1–4 times, demonstrating excellent positional repeatability of the system, absence of line broadening, and no ablation for repetitive scans.

The absence of dimensional changes of the carbon lines indicates that the observed decrease in resistance might be due to amorphous regions being converted into pyrolytic (glassy) carbon on subsequent scans as reported by others previously [30]. This effect of decreasing line resistance with an increasing number of scans is not seen for higher laser powers (Figure 6f). There, it is hypothesized that LLP is a self-limiting process in the sense that as soon as the precursor has been converted into pyrolytic carbon, the carbon line will act as a heat sink transporting the heat away from the writing spot. Apparently, this avoids super-heating of the pyrolytic carbon and thus prevents both laser-annealing and laser-ablation of the carbon unless the laser power is very high.

Further investigations into the effect of scanning the same line multiple times with different scan speeds and laser powers are summarized in Figure S5, Figure S6, Figure S7 in the Supporting Information. In conclusion, two scans were used for all subsequent optimization steps in this study where the electrical properties were of interest to maximize the percentage of conductive lines.

### 3.6. Influence of Atmosphere

**Figure 7:**
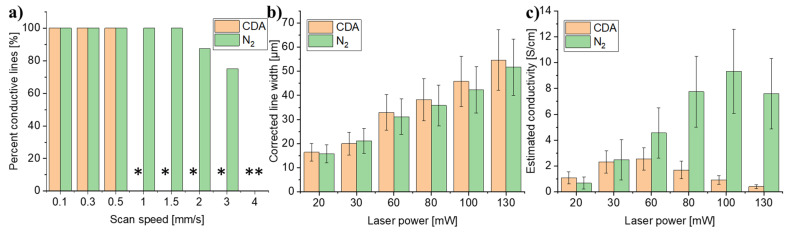
(**a**) Percentage of conductive lines (*n* ≥ 4), (**b**) corrected line width, and (**c**) estimated conductivity of lines written under different gas-purge conditions. Lines written at (**a**) 80 mW power and (**b**,**c**) 0.5 mm/s scan speed, respectively. The asterisks (*) indicate that no conductive lines were obtained at these settings. All error bars are the standard deviations (*n* = 4).

It was expected that the atmosphere would influence the writing conditions, as Mamleyev et al. [27] saw a clear influence of N_2_ saturation, both topographically, electrically, and in terms of wettability of carbon lines obtained by LLP. Therefore, the effect of an ambient air or a saturated N_2_ atmosphere was tested by purging the write-spot through the pneumatic focus nozzle in the write head with CDA and N_2_, respectively. Figure 7a shows that the N_2_ atmosphere enabled the formation of conductive lines at much higher scan speeds compared to the CDA atmosphere. Subsequently, a scan speed for which conductive lines were formed for both atmospheres was selected, and the power was varied. Figure 7b,c demonstrates that while the line width remained the same for both atmospheres, the estimated conductivity achieved was substantially higher for the lines written in the N_2_ atmosphere. This effect is attributed to an improved pyrolysis process, when the process is conducted in a saturated N_2_ atmosphere. Regular furnace pyrolysis processes likewise require an inert atmosphere [2,25,31], and the quality of the pyrolytic carbon is typically affected by the presence of O_2_ [25,41]. According to Ouyang and Hiraoka, oxidation may even cause etching (removal) of the carbon itself [41]. It might appear counterintuitive that the estimated conductivity drops for higher laser powers when exposing in a CDA atmosphere (Figure 7c). However, both Srinivasan et al. [42] and Kostecki et al. [34] observed that the carbon content declined at higher laser power. The excessive heating results in increased oxidation, which in turn results in carbon removal. Hence, all subsequent optimization steps were conducted in an inert N_2_ atmosphere.

### 3.7. Influence of Absorber Concentration

**Figure 8:**
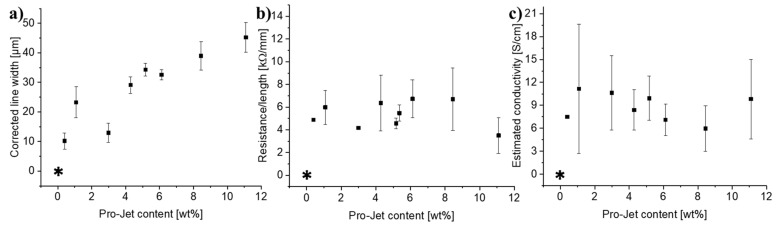
The influence of the Pro-Jet content on (**a**) corrected line width (**b**) line resistance, and (**c**) estimated conductivity. Error bars were computed using the law of error propagation (*n* = 4). The asterisks (*) signify that conductive lines could not be formed.

The effect of increasing concentrations of Pro-Jet in the SU-8 on line width and electrical properties of the pyrolytic carbon lines is shown in Figure 8. The line width increased with increasing absorber concentrations (Figure 8a) in correlation with the average recorded absorbance at 806 nm (Figure 2c). Figure 8b,c show that the line resistance and the estimated conductivity were more or less independent of the absorber concentration. For further optimization of the LLP, an absorber concentration of 5 wt % was selected as a compromise for achieving good electrical properties while keeping line-broadening low.

### 3.8. Optimization of Laser Power and Scan Speed

**Figure 9:**
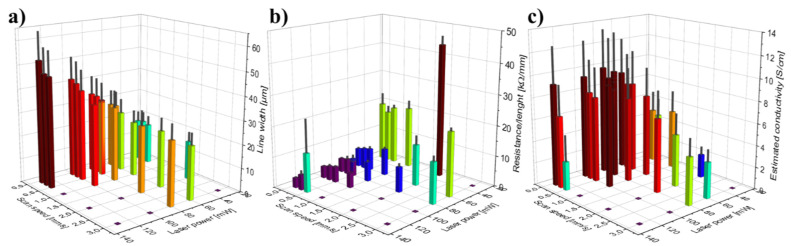
Bar plots of (**a**) corrected line width, (**b**) line resistance, and (**c**) estimated conductivity vs. scan speed and laser power (*n* = 4). The flat, purple squares indicate that conductive lines were not formed at these settings.

**Figure 10:**
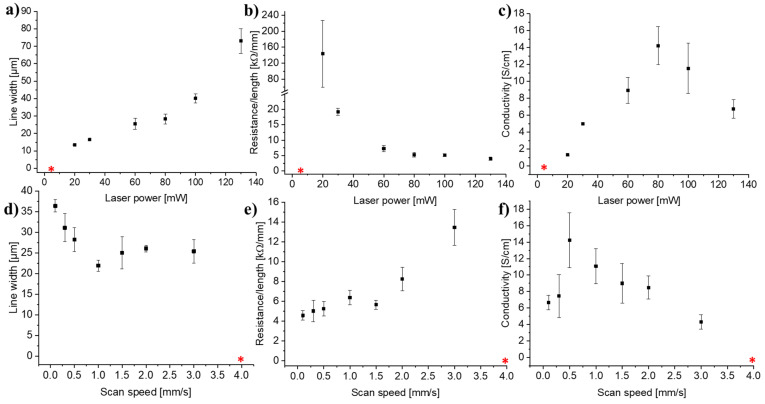
Extracts from the electrical and SEM evaluation of lines written at different laser powers and scan speeds. (**a**,**d**) actual widths of carbonized lines, (**b**,**e**) line resistances, (**c**,**f**) actual conductivities. Laser settings: (**a**–**c**) 0.5 mm/s scan speed, (**d**–**f**) 80 mW laser power. The error bars are the standard deviation (*n* = 4). The red asterisks indicate that no conductive lines were formed at these settings.

**Figure 11:**
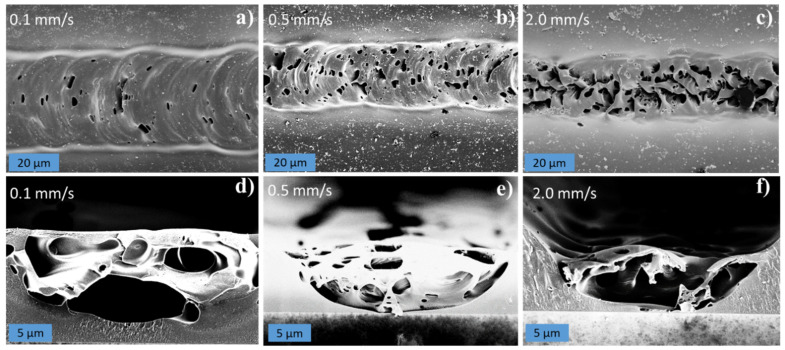
SEM images of (**a**–**c**) top view and (**d**–**f**) cross-sections of carbonized lines written with different scan speeds (power 80 mW, 2 scans). The lines in (**a**–**c**) were scanned from right to left.

The influence of laser power and scan speed on the line resistance, line width, and estimated conductivity was investigated, and the results are summarized in Figure 9. The flat, purple squares show the laser settings for which no conductive lines were formed. Figure 9a demonstrates that the line width increased for increasing laser powers and decreasing scan speeds. Figure 9b shows that the line resistance decreased for increasing laser powers and decreasing scan speeds, which was likely in part due to the increased line width seen on Figure 9a. Figure 9c indicates an optimum conductivity at medium laser powers and low scan speeds. In the investigated range, the laser power seemed to influence the width and electrical properties of the lines more profoundly than the scan speed. This is further exemplified by the graphs in Figure 10. In this detailed investigation, the actual line widths and line thicknesses were measured on SEM images such as the ones shown in Figure 11 and Figure S8 in the Supporting Information to be able to calculate more accurate values of the conductivities.

Figure 10a–c show the actual line width, line resistance, and conductivity as function of laser power at 0.5 mm/s scan speed. Figure 10a confirms that the line width increased with the laser power, while Figure 10b demonstrates that the line resistance decreased asymptotically for increasing laser powers. Figure 10c indicates that the optimal power in terms of maximizing the conductivity is 80 mW at 0.5 mm/s scan speed. The graphs in Figure 10d–f show the actual line width, line resistance, and conductivity as a function of the scan speed at 80 mW laser power. Figure 10d confirms that the line width decreased slightly with increasing scan speeds, while Figure 10e demonstrates that the line resistance increased with increasing scan speeds. Figure 10f indicates an optimal scan speed of 0.5 mm/s in terms of maximizing the conductivity at 80 mW laser power. The broadening of the lines seen for increasing laser powers and decreasing scan speeds is due to the higher absorbed energy, which in turn increases the HAZ and thus the width of the pyrolyzed region [10]. The decrease in line resistance observed for increasing laser powers and decreasing scan speeds likely has several causes: (i) The line resistance decreases with increasing line widths, which in principle is accounted for by plotting the conductivity (Figure 10c,f). (ii) The higher laser powers likely result in a higher local temperature, which in turn lead to a more conductive carbon, as observed for furnace pyrolysis and laser pyrolysis alike [23,38]. (iii) As seen on Figure 11, the scan speed affected the porosity and structural density of the carbon in the LLP lines. The increased porosity explains the higher line resistance and lower conductivity observed at high scan speeds (Figure 10e,f) because there is less conductive material available, and the material is less connected. For practical applications, the requirement of a rather low scan speed in order to achieve the better conductivity is not ideal; however, such compromises between line width, conductivity, and scan speed must be expected when dealing with laser pyrolysis. For practical applications, a hybrid approach is proposed where a high-precision laser is used for writing the small and intricate structures at low scan speed while a faster but more crude laser is used for fabricating the larger parts of the electrodes.

It is highly interesting that the porosity of the carbon lines seems to be controllable by adjusting the scan speed. In comparison, variation of the laser power had no effect on the porosity (see Figure S8 in the Supporting Information). The reason for the increased porosity with increased scan speeds is probably the more rapid heating and thus more abrupt release of gaseous by-products. In regular furnace pyrolysis, the temperature is gradually increased to ensure a controlled evaporation of non-carbonic species. Furthermore, the high temperature is typically kept stable for several hours to allow the carbon to reorganize, resulting in carbon surfaces with very low roughness and porosity [24]. However, several studies have reported increased porosity of pyrolytic carbon for faster ramping rates due to more intense degassing [43,44]. In LLP, the temperature ramping is extremely fast in comparison, regardless of scan speed, and the time that the exposed volume remains at this high temperature is extremely short. It was expected that the high porosity due to rapid heating in the LLP process should affect the conductivity of the lines compared to the slower heating in the furnace. However, the LLP process achieved a higher conductivity compared to the 3.09 S/cm previously reported for SU-8 pyrolyzed with an optimized furnace process [24]. One possible explanation for this might be that the local temperature achieved in the LLP process is significantly higher than in the furnace process, resulting in more conductive pyrolytic carbon. Unfortunately, a direct measurement of the local temperature during writing was impossible in our current system.

The groove depth was also affected by the scan speed and laser power. A more in-depth study of the effect of various laser parameters on the groove depth can be found in Figure S6a and Figure S7a in the supporting information. In general, the groove depth increased with increasing laser power and decreasing scan speeds but remained unaffected by the number of repetitive scans. The thickness of the lines remained constant (see Figure S3a,d and Figure S7c in the Supporting Information).

The line in Figure 11b,e was scanned twice at 80 mW laser power and 0.5 mm/s scan speed. The thickness of the pyrolyzed line on the SEM image in Figure 11e was 6.0 ± 1.0 µm, and the width was 28.3 ± 2.9 µm. The measured line resistance was 5.2 ± 0.7 kΩ/mm. The resultant conductivity was 14.2 ± 3.3 S/cm, assuming a semi-elliptical cross-section. This is more than four times better than the conductivity of 3.09 S/cm previously reported for furnace pyrolyzed SU-8 [24]. The smalslest line width obtained for a conductive line was 13.5 ± 0.4 µm wide and 4.9 ± 0.5 µm thick, achieved at 0.5 mm/s scan speed and 20 mW laser power (see Figure 10b), however, it came at the expense of a higher line resistance of 143.7 ± 83.4 kΩ/mm and a lower conductivity of 1.3 ± 0.2 S/cm. In Table 1, these values are compared with conductivities and line dimensions reported in other works on LLP. The pyrolytic carbon produced by laser pyrolysis of SU-8 reaches conductivities similar to 10–20 S/cm reported by most other studies in the field focusing on polyimide as a polymer precursor. This is comparable even to the conductivities of some LIG films [5,45]. Furthermore, we demonstrate a higher resolution compared to the CO_2_-lasers [27,46]. Our resolution is only surpassed by the KrF and Ti:Sapphire lasers, for which the reported conductivities generally are lower than the values reported here [47,48].

Compared to LLP lines written with the commercially available CO_2_ laser (see Figure S9 in the Supporting Information), the lines written with our customized system are substantially thinner. The CO_2_ laser enables faster writing of pyrolytic carbon lines; however, it does not discriminate between SU-8 films with and without the Pro-Jet included. From Figure 10, it seems that the optimum conductivity is achieved at 80 mW power and 0.5 mm/s scan speed. However, as the variance was much lower for lines written at 80 mW power and 0.1 mm/s scan speed, these settings were used for the subsequent analysis of complex structures.

### 3.9. Lines, Intersects, and Joints

**Figure 12:**
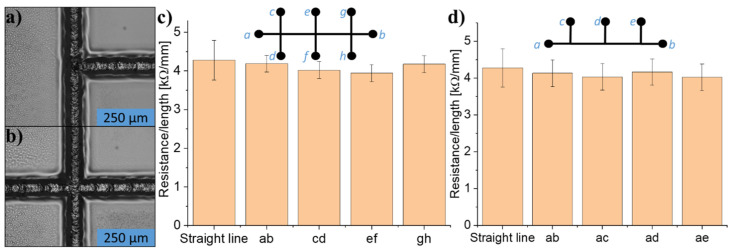
(**a**,**b**) show the joining and intersecting of two laser-pyrolyzed lines. The vertical lines are written first, the horizontal lines are written second. (**c**,**d**) show resistance per path length measured between the points shown on the schematic insets compared to the resistance of a straight line written with the same parameters. All lines were written at 80 mW power, 0.1 mm/s scan speed, using two scans. The error bars are based on the standard deviation (*n* ≥ 3).

The resistance increased linearly for lines with different lengths (see Figure S10a in the Supporting Information). Furthermore, seamless joining and intersecting of lines, as presented in Figure 12a,b, was possible. This seamless joining is confirmed electrically in Figure 12c,d. The lines were joined (Figure 12a) or intersected (Figure 12b) by first writing one vertical line and then writing a horizontal line, either joining or crossing with the vertical one. This can be done several times as illustrated on the inset schematics in Figure 12c,d. The resistance was measured between all end-points (*a, b,…, h)* on the test patterns (see inset schematics on Figure 12c,d) and normalized to the path length between the two particular end-points. The weighted average resistance per length was compared to the resistance per length obtained from measurements of single straight lines. As seen, there is no difference in the resistance per length obtained, regardless of the test pattern or the two selected end-points. Furthermore, the direct extension of an existing line in a completely seamless manner was also possible, as shown in Figure S10b in Section S7 of the Supporting Information.

### 3.10. Laser Writing Line Resolution

**Figure 13:**
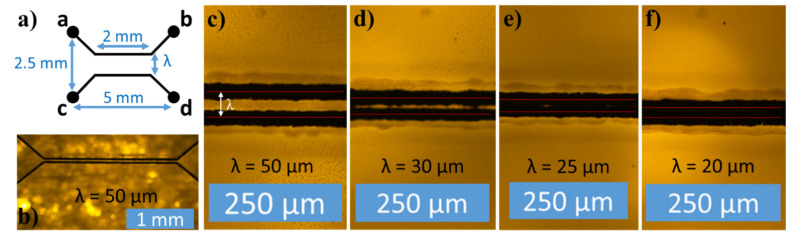
(**a**) Schematic of test structure, (**b**) image of test structure**,** (**c**) lines insulated, (**d**) lines still insulated, (**e**) insulation of lines starts to break down, (**f**) insulation of lines completely broken down. The red lines indicate the center of the beam trace with λ being the pitch between the two trace centers. All lines were written at 80 mW and 0.1 mm/s scan speed.

Finally, the line resolution of the direct laser writing process was investigated. More specifically, the minimal spacing between two lines without electrical short circuit was identified. For this purpose, two parallel lines were written with decreasing center-to-center distance (pitch) *λ*, as shown in Figure 13a,b. The two lines branch out and away from each other at the ends to allow space for the contacts and electrical probes. Resistance was measured between all end-points to check if the two parallel lines were insulated from each other while confirming that neither of the two parallel lines were broken. It was found that the lines were electrically insulated from each another for *λ* ≥ 30 µm (Figure 13c,d). At *λ* = 25 µm (Figure 13e), the insulation started to break down, and a resistance about 10 times larger than for a fully connected line could be measured. At *λ* = 20 µm, there was a full short circuit between the parallel lines and the measured resistance corresponded to the one measured through a single, conducting line. The fact that there was no short circuit until *λ ≤* 25 µm, is in agreement with the actual line widths of carbonized lines of approximately 30 µm discussed above. The actual gap *g* between the lines on Figure 13d is approximately 5 µm.

## 4. Conclusions

We have demonstrated a novel method for the selective and direct laser writing of conductive carbon lines in SU-8 by the inclusion of a wavelength-specific absorber. SU-8 is locally pyrolyzed by adding an absorber to the resin before spin-coating and subsequently irradiating it with a focused, NIR, semiconductor-diode, CW laser. Direct laser writing in inert nitrogen atmosphere resulted in higher conductivity. In general, a higher laser power and a lower scan speed resulted in larger line width and lower line resistance.

The maximum conductivity achieved with the optimized laser writing was 14.2 ± 3.3 S/cm, which is four times better than the one obtained for optimized furnace pyrolysis of SU-8 [24]. The minimum line width achieved for a conductive line was 13.5 ± 0.4 µm with the compromise of an increased line resistance and lower conductivity of the pyrolytic carbon. Laser pyrolysis of SU-8 by means of a commercially available CO_2_-laser was indeed possible. However, the CO_2_-laser produced substantially wider lines and did not discriminate between SU-8 with and without the absorber. Additionally, the porosity of the lines could be controlled by adjusting the scan speed of our laser. The highly porous structures obtained at higher scan speeds could be very beneficial for applications such as electrochemical energy storage or biosensing. As for the inclusion of the Pro-Jet 800NP, we believe that the ability to pyrolyze only the selected, absorber-modified regions may hold a lot of potential for device fabrication as well as the selective pyrolysis of other polymer precursors.

## Figures and Tables

**Table 1:** Table of reported conductivities and resolutions achiesved through LLP using various laser typses on various substrates.

Year	Precursor Material	Laser	Conductivity [S/cm]	Smallest Reported Conductive Line Width [µm]	Smallest Reported Conductive Film Thickness [µm]	Reference
1991	Polyimide	KrF laser	10	NA	NA	[49]
1993	Polyimide	KrF laser	10	NA	NA	[50]
1993	Polyimide	KrF laser	2	0.5	NA	[47]
1994	Polyimide	Ar ion	25	15	NA	[42]
2002	OiR 897-101 positive photoresist	HeNe and Ar-ion lasers	NA	20	NA	[34]
2004	π-conjugated polyaniline emeraldine base	C^2+^, F^2+^, and Cl^2+^ ions (5SDH-2 tandem Van de Graff accelerator)	60	NA	6.2	[51]
2012	Graphite oxide films	LightScribe DVD burner	16.5	500	NA	[45]
2013	Graphite oxide films	LightScribe DVD burner	23.5	20	7.6	[11]
2014	Polyester	Ti:Sapphire laser	0.3	2	NA	[48]
2014	polyimide	CO_2_ laser	25	NA	23.4	[38]
2015	Polyimide	Pulsed, femtosecond fiber laser	6	40	18	[10]
2016	Polyimide	CO_2_ laser	11	110	30	[5]
2017	Polyimide	CO_2_ laser	15.4	200	31	[8]
2017	Polyimide	CO_2_ laser	0.2	15	50	[46]
2019	Polyimide	CO_2_ laser	25	25	25	[27]
2020	SU-8	CW semiconductor diode laser (80 mW, 0.5 mm/s, 2 scans, N_2_ atmosphere)	14.2 ± 3.3	28.3 ± 2.9	6.0 ± 1.0	This work
2020	SU-8	CW semiconductor diode laser (20 mW, 0.5 mm/s, 2 scans, N_2_ atmosphere)	1.3 ± 0.2	13.5 ± 0.4	4.9 ± 0.5	This work

## Data Availability

Data will be available from 01.06.2021 at https://data.dtu.dk/projects/PHOENEEX/96182

## Supplementary Materials

The following are available online at https://www.mdpi.com/article/10.3390/mi12050564/s1, Figure S1: Overview of all recorded absorbance spectra for wafers coated with various concentrations of Pro-Jet. All spectra were recorded vs. air; Figure S2: (a) XPS-spectrum of absorber-modified SU-8 prior to LLP. In-depth XPS analysis of a carbon line. (b) Oxygen content vs. etching depth (sputter time). (c) Carbon content vs. etching depth (sputter time); Figure S3: (a) and (d) line thicknesses obtained by cross-sectional SEM imaging of lines written with different laser powers (at 0.5 mm/s scan speed) and scan speeds (at 80 mW laser power). (b) and (e) line widths obtained by OM and SEM imaging for different laser powers at different scan speeds. (c) and (f) ratios of OM line width to SEM line width, revealing a consistent overestimation by OM; Figure S4: (a) and (b) show respectively a cross-section and a 3D image of the topography of a pyrolysed line. (c) and (d) show respectively a cross-section and a 3D image of the topography of an ablated line where pyrolysis was not achieved. This line will not be conductive; Figure S5: Percentage of conductive lines (out of 4) for (a) 1 scan and (b) 2 scans at different scan speeds and laser powers. The flat, purple squares indicate that no conductive lines were formed at these settings; Figure S6: (a) groove depth, (b) corrected line width, and (c) line resistance vs. number of scans and laser power at different scan speeds. The error bars correspond to the standard deviation (n = 4); Figure S7: (a) depth of ablated groove, (b) actual width of carbonized line, and (c) thickness of carbonized line vs. number of scans. The error bars correspond to the standard deviation with (n ≥ 3); Figure S8: SEM images of (a–c) the tops and (d–f) cleaved cross-sections of lines written with different powers at 0.1 mm/s scan speed. As seen, the lines are very similar, except that they get wider; Figure S9: (a) Line resistance, (b) line width, and (c) estimated conductivity vs. scan speed for lines written with the CO_2_-laser at 3 W power on a wafer containing 5.17 wt % Pro-Jet. The error bars are the standard deviation (n = 4). Insert on a) LLP lines written by a CO_2_-laser in clear SU-8 coated on a boronglass wafer. Inset on c) is a line written with 1.5 W power and 25 mm/s scan speed, the scale bar is 1 mm; Figure S10: (a) Resistance vs. path length. (b) A resistor made by lateral joining of two lines written with 80 mW (left part) and 30 mW (right part) laser power respectively at 0.5 mm/s scan speed. The schematic of the full resistor structure is shown on the inset (top). An equivalent circuit model for calculating the theoretical resistance through the structure is also seen on the inset (bottom) where *R*_1_ = *R*_3_. (c) Table of the theoretical and measured resistances through the resistor shown on (b). As seen, the difference is very small.
