# Supplementary material for: Selective Direct Laser Writing of Pyrolytic Carbon Microelectrodes in Absorber-Modified SU-8
Source: Micromachines (Basel). 2021 May 17;12(5):564. doi: 10.3390/mi12050564 (PMC8156660; doi:10.3390/mi12050564)
Supplement: Supplementary file 1 [file micromachines-12-00564-s001.zip › micromachines-1211813-supplementary.pdf]

Selective Direct Laser Writing of Pyrolytic Carbon Microelectrodes in Absorber-Modified SU-8

Emil Ludvigsen, Nina Ritter Pedersen, Xiaolong Zhu, Rodolphe Marie, David M. A. Mackenzie, Jenny Emnéus, Dirch Hjorth Petersen, Anders Kristensen and Stephan Sylvest Keller

Supporting information

S.1. Recorded absorption spectra

In figure S1, all the recorded absorption spectra for wafers coated with SU-8 containing various concentrations of Pro-Jet can be seen. The spectra were recorded vs. air from 200-1100 nm, however, only the range from 450-900 nm is shown here, as this was where the Pro-Jet had its effect. A saturation is seen in the 750-800 nm range for Pro-Jet contents >4 wt%.

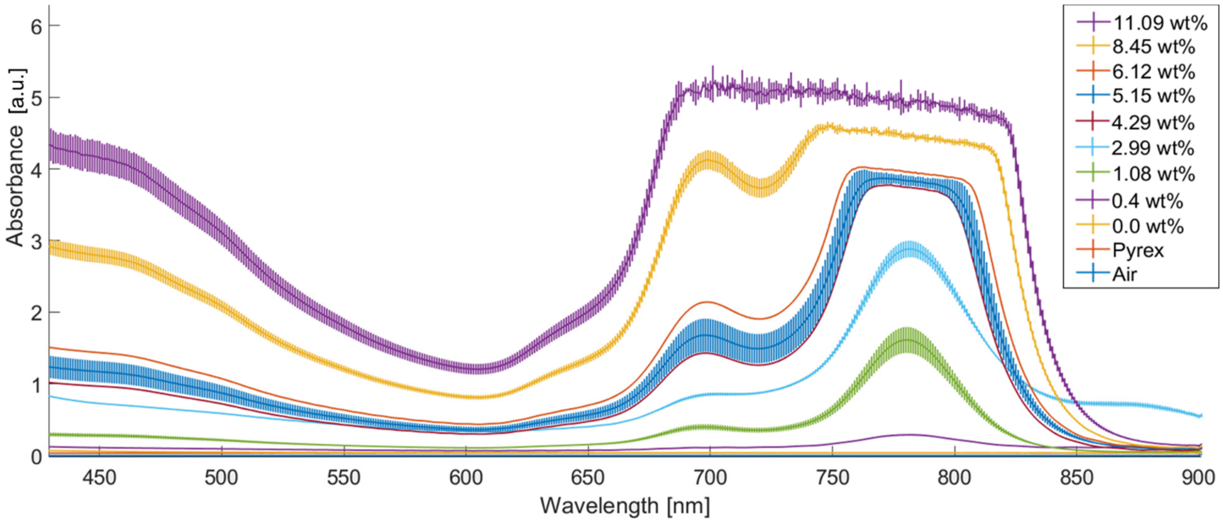

Figure S1: Overview of all recorded absorbance spectra for wafers coated with various concentrations of Pro-Jet. All spectra were recorded vs. air.

S.2. Chemical evaluation of carbon lines

Figure S2 a) shows the XPS-spectrum of absorber-modified SU-8 prior to LLP. It looks a lot like the XPS-spectrum for the surface of an LLP line (see figure 4 b)), apart from the presence of an F1s peak that is absent on figure 4 b). Figure S2 b) and c) show the in-depth XPS-analysis of the oxygen (O1s) and carbon (C1s) contents respectively, as the Ar-ions mill deeper into the carbonized line. Each cycle is 5 s of Ar-ion sputtering, and removes a few nm of material. 9 cycles, i.e. a total of 45 s of sputtering was done, and an XPS-spectrum was recorded after each 5 s cycle. As seen, only the very top-layer of the carbon is oxidized, whereas the deeper layers reveal almost exclusively sp^2-hybridized carbon.

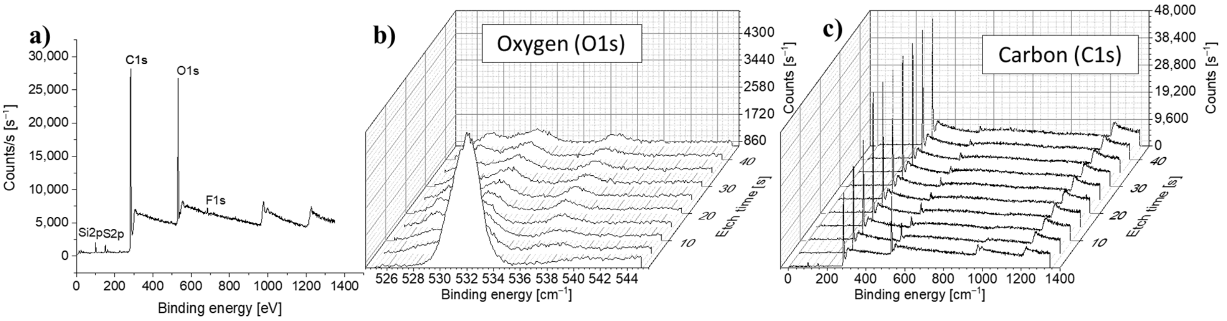

Figure S2: **(a)** XPS-spectrum of absorber-modified SU-8 prior to LLP. In-depth XPS analysis of a carbon line. **(b)** Oxygen content vs. etching depth (sputter time). **(c)** Carbon content vs. etching depth (sputter time).

S.3. Width adjustment for OM images

As mentioned, it was found that the line widths obtained from the OM images consistently overestimated the actual width of the carbonized lines by an average factor of 4.17±0.94. The cause of the overestimation is that the full line width of the blackened line was used, not just the central, carbonized part which was not resolvable on the regular OM images. Thus, the widths obtained from the OM images were reduced by a factor of 4.17. The combined, relative error was 22.5%. The error on the reduction factor has been accounted for in accordance with the law of error propagation, by treating the reduction factor $F \pm \delta F$ as an independent parameter. The adjusted width, $w_{adj} \pm \delta_{adj}$, has then been calculated as in equation S1 and equation S2.

$$w_{adj} = \frac{w_{OM}}{F} = \frac{w_{OM}}{4.17} \tag{S1}$$

$$\delta_{adj} = \sqrt{\left(\frac{\delta_{OMW}}{F}\right)^2 + \left(\frac{\delta F}{F} \cdot \frac{w_{OM}}{F}\right)^2} = \sqrt{\left(\frac{\delta_{OMW}}{4.17}\right)^2 + \left(0.2245 \cdot \frac{w_{OM}}{4.17}\right)^2} \tag{S2}$$

Where w_{adj} is the average adjusted width. w_{OM} is the average width measurement obtained from OM. δ_{adj} is the combined adjusted error on the adjusted average width. δ_{OMW} is the standard deviation on the average width measurement obtained from OM. $F = 4.17$ is the reduction factor, i.e. the overestimation factor from OM width measurements, compared to the actual widths obtained from cross-sectional SEM imaging. $\delta F = 0.94$ is the error on the reduction factor and $\frac{F}{\delta F} = 0.225$ is the combined, relative error of the widths obtained from SEM imaging and the widths obtained from OM imaging.

Extracted plots of the widths obtained by SEM- and OM imaging can be seen in figure S3 b) and e) for different laser powers at different scan speeds. The factor $\frac{w_{OM}}{w_{SEM}}$ has likewise been plotted in figure S3 c) and f). Furthermore, from figure S3 a) and d) it is evident, that the thickness of the carbonized line is more or less constant, independently of the exposure parameters used.

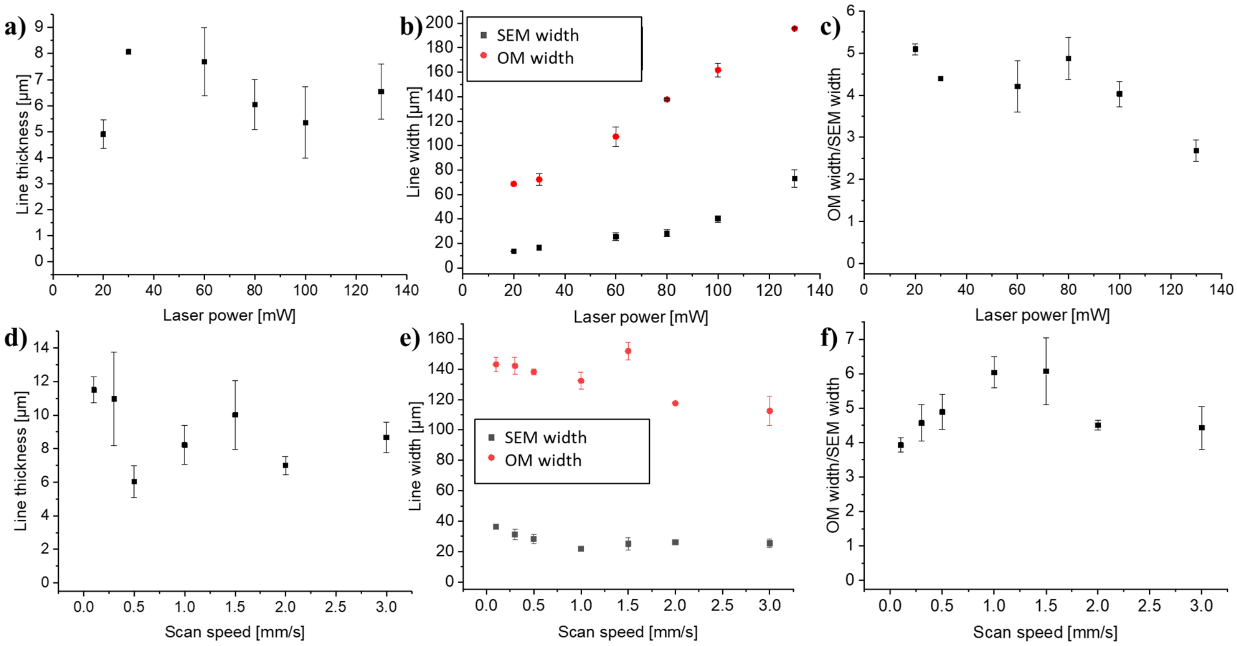

Figure S3: **(a)** and **(d)** line thicknesses obtained by cross-sectional SEM imaging of lines written with different laser powers (at 0.5 mm/s scan speed) and scan speeds (at 80 mW laser power). **(b)** and **(e)** line widths obtained by OM and SEM imaging for different laser powers at different scan speeds. **(c)** and **(f)** ratios of OM line width to SEM line width, revealing a consistent overestimation by OM.

S.4. 3D profiles of laser written lines

Topographical profiles, obtained by optical profilometry of a carbonized and a non-carbonized line, can be seen in figure S4 a-b) and c-d) respectively. The height of the roughness protrusions in the bottom of figure S4 b) are most likely exaggerated. The kink in the middle of figure S4 c) and d) is a true property of non-pyrolysed lines as it was confirmeds by stylus profilometry. The kink probably arises due to the generated heat causing a melting of the polymer and an expansion of gasses. However, the heat is not great enough to burst the bubble and form the porous, carbon seen for fully pyrolysed lines.

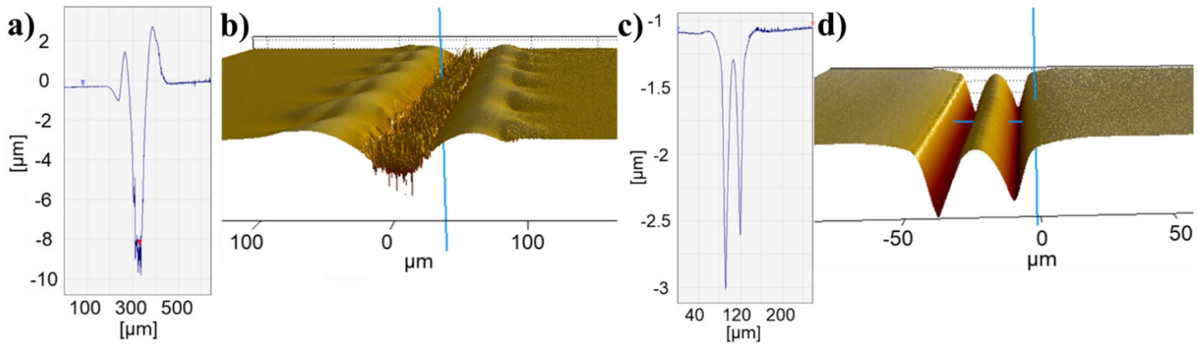

Figure S4: **(a)** and **(b)** show respectively a cross-section and a 3D image of the topography of a pyrolysed line. **(c)** and **(d)** show respectively a cross-section and a 3D image of the topography of an ablated line where pyrolysis was not achieved. This line will not be conductive.

S.5. Influence of laser settings

Figure S5 a) and b) show the percentage of conductive lines (out of four) achieved for various scan speeds and laser powers at one and two scans respectively. As seen, the percentage of conductive lines is significantly improved when going from one to two scans of the same line.

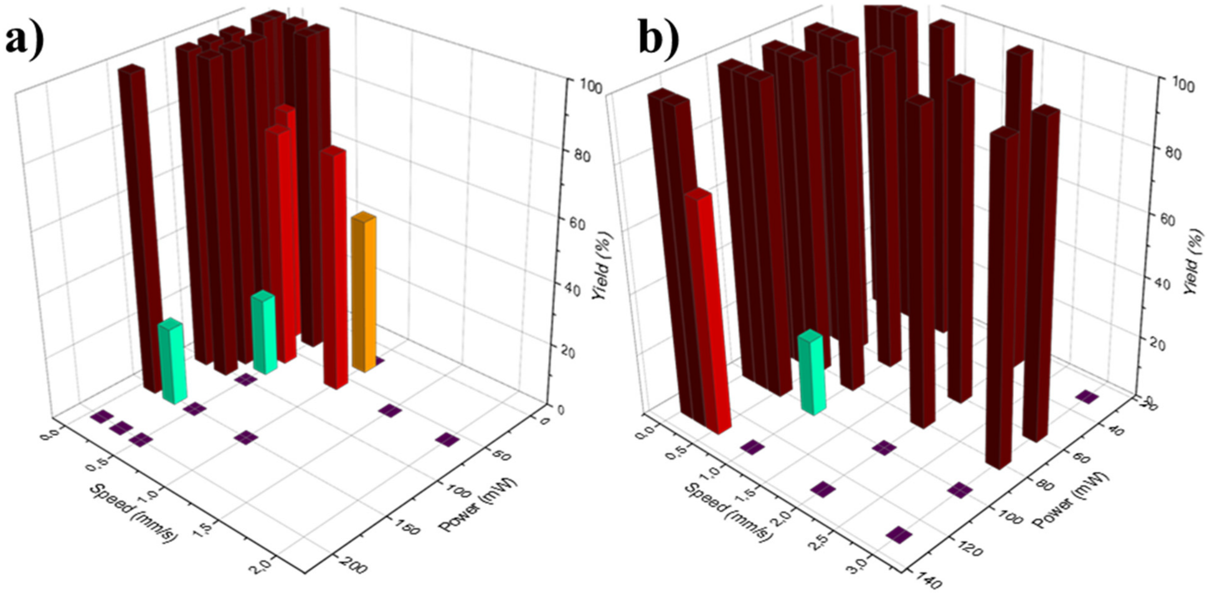

Figure S5: Percentage of conductive lines (out of 4) for **(a)** 1 scan and **(b)** 2 scans at different scan speeds and laser powers. The flat, purple squares indicate that no conductive lines were formed at these settings.

Figure S6 shows the groove depth, corrected line width, and line resistance as function of laser power and the number of scans of the same line for various scan speeds. Figure S7 shows the groove depth, actual line width, and line thickness of the carbonized lines, written at various laser powers for one and two scans of the same line. The graphs in figure S6 have been achieved by a combination of optical profilometry and OM, while the graphs in figure S7 has been

achieved by cross-sectional SEM imaging. The widths in figure S6 b) have been reduced according to the method described in section S.1. of the supporting information. The depth of the ablated groove as function of number of scans was measured to check that the multiple scans did not result in ablation/removal of the already carbonized lines, The results can be seen in figure S6 a) and figure S7 a). The groove depths obtained from the two techniques seem to agree completely. No additional ablation was observed when going to multiple scans, as the groove depth remained the same from scan to scan. As seen on figure S6 b) and figure S7 b), there is little-to-no increase in the line width when scanning the same line multiple times. The same goes for the thickness of the pyrolysed part of the lines (see figure S7 c)). A slight improvement of the line resistance can be seen from one to two scans (see figure S6 c)), however, there is no further improvement of the line resistance for more than two scans of the same line. The data in figure S6 c) is merely an expansion to more laser powers of the data presented in figure S6 g). Since the line width, and line thickness remained independent of the number of scans of the same line, the improved line resistance can be attributed to one of two things. Either an improved pyrolysis process resulted in more graphitic carbon due to laser annealing on the second scan. Or the improved line resistance is a result of partly- or non-carbonized micro-sections being "repaired" by the second scan as observed for macro-sections in figure 6 c), thus forming a less disjointed line. The latter might be more likely as the groove depth did not increase either, for an increasing number of scans. This could suggest that the laser is not super-heating the already-pyrolysed carbon regions since material removal and thus a deeper groove would be expected for multiple repeated scans if super-heating took place.

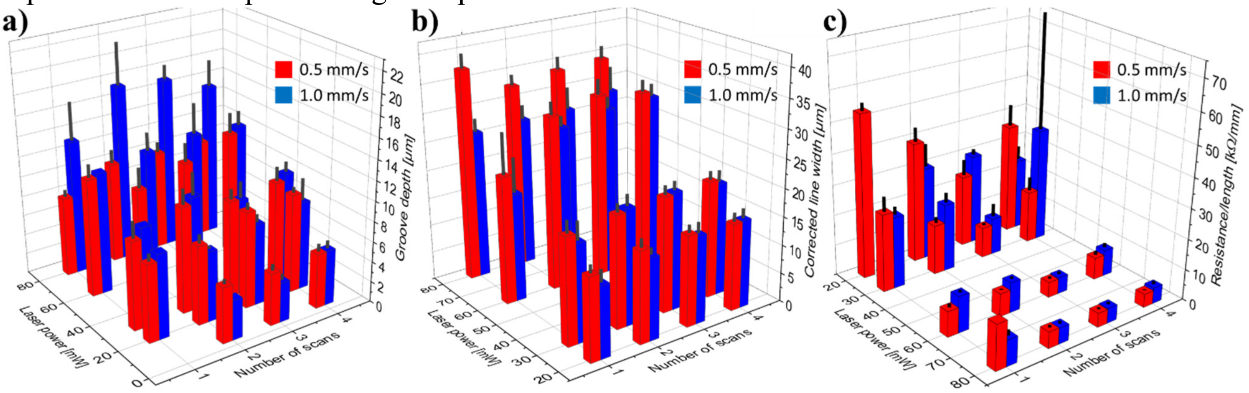

Figure S6: **(a)** groove depth, **(b)** corrected line width, and **(c)** line resistance vs. number of scans and laser power at different scan speeds. The error bars correspond to the standard deviation (n=4).

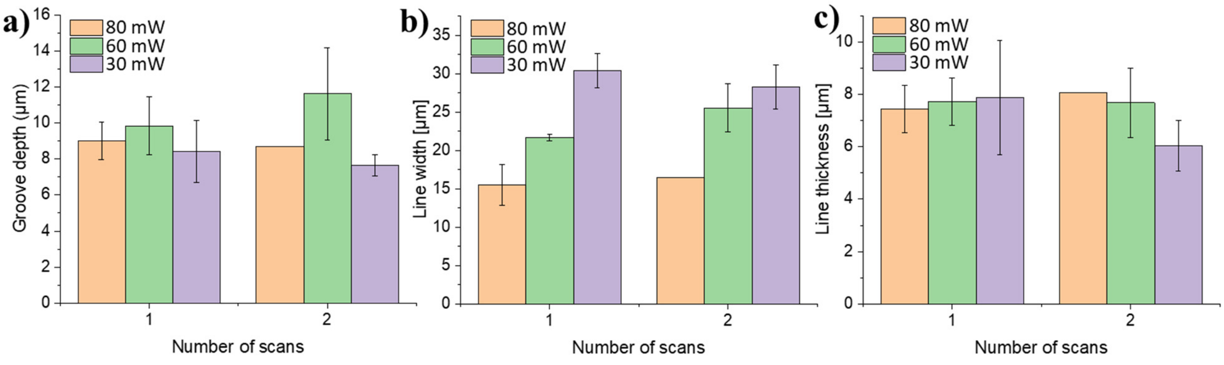

Figure S7: **(a)** depth of ablated groove, **(b)** actual width of carbonized line, and **(c)** thickness of carbonized line vs. number of scans. The error bars correspond to the standard deviation with (n≥3).

SEM images of the tops and cross-sections of lines, written with different laser powers at 0.1 mm/s scan speed can be seen in figure S8. It is from images such as these, that the actual line widths and line thicknesses have been extracted.

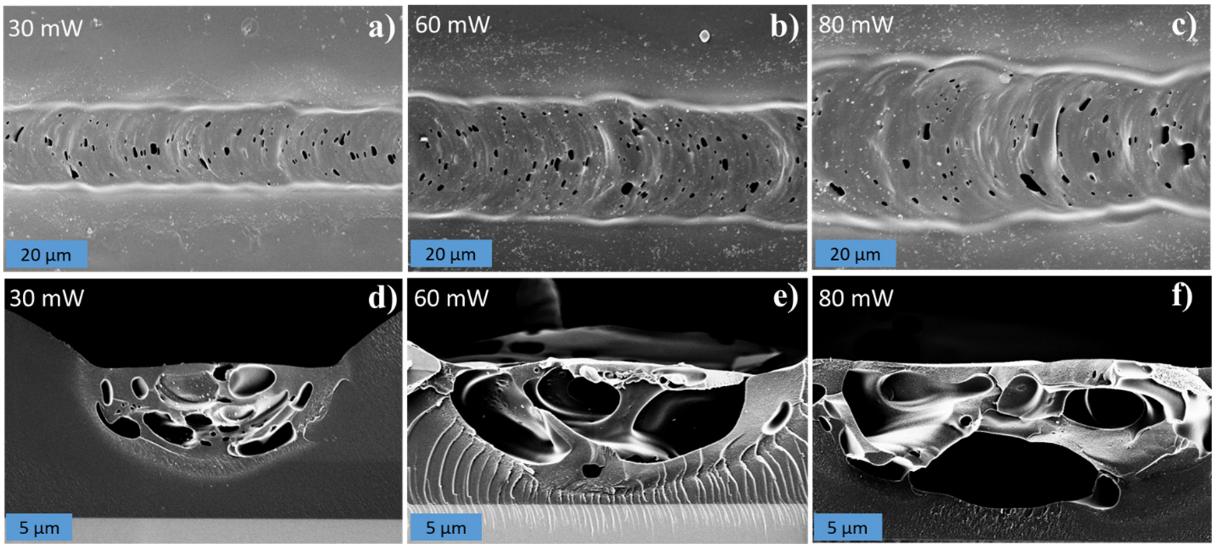

Figure S8: SEM images of **(a-c)** the tops and **(d-f)** cleaved cross-sections of lines written with different powers at 0.1 mm/s scan speed. As seen, the lines are very similar, except that they get wider.

S.6. Local laser pyrolysis by commercially available CO_2-laser

A commercially available, pulsed CO_2-laser (Epilog Helix Mini 18, EpilogLaser) operating at 10.6 µm wavelength with a spot size of 100 µm in diameter was tested, in order to see if LLP of SU-8, both with and without absorber, was possible with a different laser. It was indeed possible to pyrolyse both the clear and the absorber-modified SU-8 using the CO_2-laser. On the inset on figure S9 a) four distinct, blackened (carbonized), laser-pyrolysed lines can be seen. The lines were written by the CO_2-laser in clear SU-8 2005 resin coated on a boronglass wafer. The distance between the lines is 3 mm and the length of each line is 5 mm. The presence of these lines clearly

show that pyrolysis of pure SU-8 is indeed possible, as long as the operating window for the laser beam is within the absorption range of SU-8. This image should be compared with figure 3 b), which shows that the 806 nm laser of our modified mask-less aligner system is not absorbed by the clear SU-8 and thus require the inclusion of Pro-Jet in the resin, in order for LLP to work. The resistance per length of the lines shown on the inset of figure S9 a) was 384.2±54.4 Ω/mm, and the width was 333.3±0.1 µm. This confirms that the increase in conductance from the LLP treatment is due to the carbonization of the polymer, rather than the sintering of any metallic particles that may be present in the Pro-Jet. The CO_2-laser was also employed to write lines on a wafer coated with the absorber-modified SU-8. The results of this study can be seen on the graphs of figure S9 a-c). The exposure parameters were 10% power (=3 W) and 10% scan speed (=10 mm/s) using just one scan. The exposure was done while purging the writing spot with N_2 from the built-in N_2-gun. Due to the higher power and wider spot size, the written lines became substantially wider (see figure S9 b)). Comparing figure S9 a-c) to the graphs of figure 10 d-f), we see the same trends but a lower estimated conductivity, probably due to the CO_2-lasing process not being optimized. It was seen that for the CO_2-laser at high powers or low speeds the central parts of the carbon lines had been completely ablated. This left a carbon-free trace in the middle of the carbonized path (see inset on figure S9 c)), thus only the width of the black part of the lines were used in the estimation of the line width.

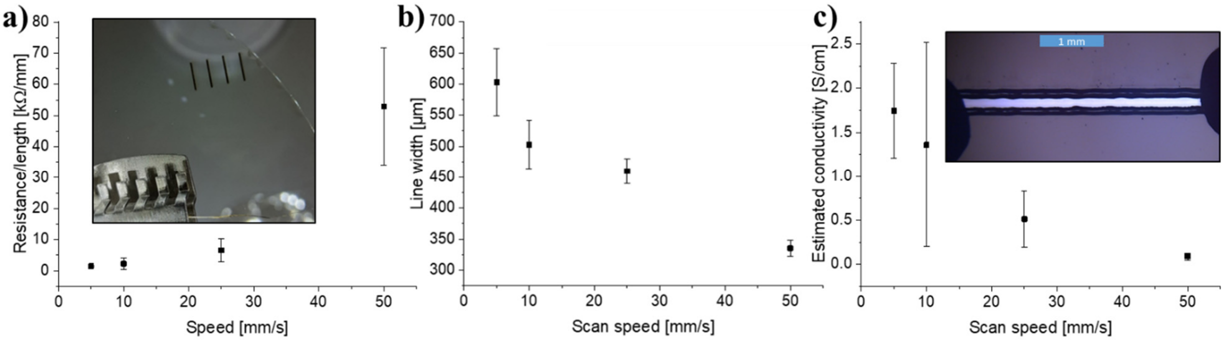

Figure S9: **(a)** Line resistance, **(b)** line width, and **(c)** estimated conductivity vs. scan speed for lines written with the CO_2-laser at 3 W power on a wafer containing 5.17 wt% Pro-Jet. The error bars are the standard deviation (n=4). Insert on a) LLP lines written by a CO_2-laser in clear SU-8 coated on a boronglass wafer. Inset on c) is a line written with 1.5 W power and 25 mm/s scan speed, the scale bar is 1 mm.

S.7. Joining and intersecting lines

In figure 12 a-c) we showed that joining and intersecting lines occurred in a seamless manner. Here, we show that the resistance increases with the path length in a highly linear manner (figure S10 a)) and that integrated circuit components, such as resistors can be drawn directly by changing the laser power and joining lines laterally (figure S10 b)). The resistance through the resistor was measured and compared to the theoretically expected resistance through the structure (see table in figure S10 c)). As seen, the measured resistance is very close to the theoretically predicted resistance through the structure. The total theoretical resistance R_{tot} through the structure was calculated as,

$$R_{tot} = R_1 + R_2 + R_3 = \frac{R_{80}}{L_{80}}L_1 + \frac{R_{30}}{L_{30}}L_2 + \frac{R_{80}}{L_{80}}L_3 \qquad (S3)$$

Where the resistances R_{80} and R_{30} are the measured resistances through straight lines written with 80 mW and 30 mW laser power respectively at 0.5 mm/s scan speed. L_{80} and L_{30} are the lengths of the straight lines through which R_{80} and R_{30} were measured. L_1, L_2, and L_3 are the lengths of each resistor part, R_1, R_2, and R_3 shown on the equivalent circuit schematic on the lower part of figure S10 b), with $L_1 = L_3$.

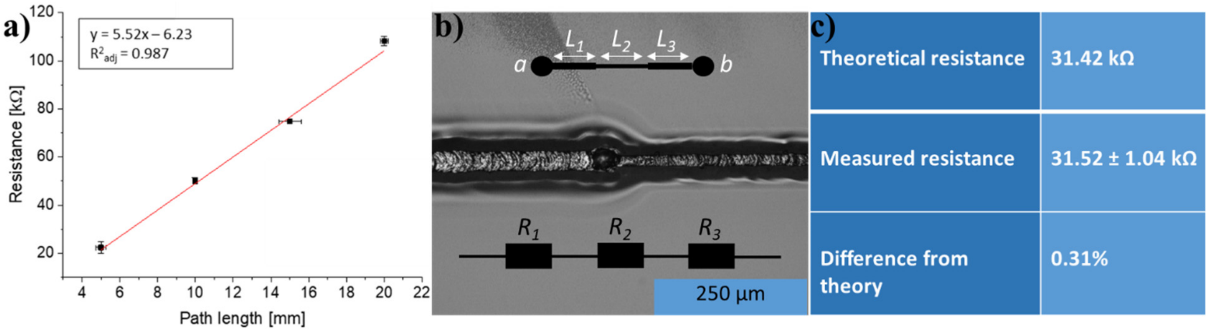

c)

Theoretical resistance	31.42 kΩ
Measured resistance	31.52 ± 1.04 kΩ
Difference from theory	0.31%

Figure S10: **(a)** Resistance vs. path length. **(b)** A resistor made by lateral joining of two lines written with 80 mW (left part) and 30 mW (right part) laser power respectively at 0.5 mm/s scan speed. The schematic of the full resistor structure is shown on the inset (top). An equivalent circuit model for calculating the theoretical resistance through the structure is also seen on the inset (bottom) where $R_1 = R_3$. **(c)** Table of the theoretical and measured resistances through the resistor shown on b). As seen, the difference is very small.
